# Impact of ZnO Modifier Concentration on TeO_2_ Glass Matrix for Optical and Gamma-Ray Shielding Capabilities

**DOI:** 10.3390/ma15155342

**Published:** 2022-08-03

**Authors:** Dalal A. Aloraini, M. I. Sayyed, Ashok Kumar, Sabina Yasmin, Aljawhara H. Almuqrin

**Affiliations:** 1 Department of Physics, College of Science, Princess Nourah Bint Abdulrahman University, P.O. Box 84428, Riyadh 11671, Saudi Arabia; daalorainy@pnu.edu.sa (D.A.A.); ahalmoqren@pnu.edu.sa (A.H.A.); 2 Department of Physics, Faculty of Science, Isra University, Amman 11622, Jordan; 3 Department of Physics, University College Benra, Dhuri 148024, Punjab, India; ajindal9999@gmail.com; 4 Department of Physics, Punjabi University, Patiala 147002, Punjab, India; 5 Department of Physics, Chittagong University of Engineering and Technology, Chattogram 4349, Bangladesh; sabinayasmin309@gmail.com

**Keywords:** optical dielectric constant, ZnO, transmission coefficient, low energy

## Abstract

This study carried out a comparison of the optical and gamma ray shielding features of TeO_2_ with and without ZnO modifier concentration. Incorporating ZnO into the TeO_2_ network reduces the indirect band gap from 3.515–3.481 eV. When ZnO is added, refractive indices, dielectric constants, and optical dielectric constants rise from 2.271–2.278, 5.156–5.191, and 4.156–4.191 accordingly. The transmission coefficient and reflection loss are in direct opposition to each other. With increasing ZnO concentration in the selected glasses, the values of molar refractivity and molar polarizability decrease from 18.767–15.018 cm^3^/mol and from 7.444 × 10^−24^–5.957 × 10^−24^ cm^3^, respectively, while the electronic polarizability rises from 8.244 × 10^24^–8.273 × 10^24^, correspondingly. As expected by the metallization values, the glass systems are non-metallic. The linear attenuation coefficients (LAC) of the studied glass samples ensue through enhancing the photon energy range 0.0395–0.3443 MeV. There is a very slow decrease in the LAC from an energy of 0.1218–0.3443 MeV, yet there is a sharp decrease from an energy of 0.0401–0.0459 MeV. According to the obtained values of numerous shielding parameters such as LAC, MAC, HVL, MFP, and Z_eff_ sample, Zn30 has shown the best radiation shielding ability comprising other studied samples.

## 1. Introduction

With the remarkable development in radiation technology, researchers’ attention has been focused on radiation shielding materials. These materials are utilized for a variety of industrial and radiologic applications. They are frequently used to attenuate the intensity of the radiations and thus help in protecting the workers, patients, and people from the ionizing radiation. Lead is one of the first materials utilized to manufacture radiation shielding materials [1,2,3,4]. The high density of lead has made it a promising shielding material until now. The intensive studies carried out in the radiation shielding materials field have found that lead has some disadvantages, and for this reason, radiation shielding material technologists have produced alternative free lead radiation shielding materials [5,6,7,8,9,10]. Among these novel materials, glasses have many advantages and possess unique features that encourage those interested in this field to develop novel glass systems in different shielding applications [11,12,13]. These unique features include the simplicity in synthesis of the glasses with low cost, the good mechanical properties of the glasses, the high transparency, and the high density of the glasses in comparison with other materials such as polymer, concrete, and bricks [14,15]. These outstanding features make the glasses ideal to be utilized for radiation protection applications. Due to the high possibilities in various fields, the continuous attempts in new glass systems and the investigation of their radiation attenuation competence are needed. TeO_2_-based glasses have received notable attention from radiation shielding developers since they have a low melting point, good mechanical strength, high refractive index, high density, high effective atomic number, low toxicity, and low tenth value layer [16,17]. Aşkın et al. have studied the gamma shielding potential of the newly established glass system through the Geant4 code. At energy ranges 0.284, 0.356, 0.511, 0.662, 1.17,3, and 1.330 MeV, Tellurite glass containing 25% moles of Ag_2_O showed the maximum mass attenuation coefficient and Z_eff_, however, the lowermost value of transmission fractions. The fast neutron removal cross-section (∑R) of the studied glasses amplified with the upsurge of Ag_2_O fraction as well as the ∑R values were greater than commonly used concrete material but a close value to the NiO and PbO-containing borate glasses [18]. Sharma et al. investigated zinc tellurite glasses doped with Nd_2_O_3_. Having the greatest percentage of Nd_2_O_3_ (0.05) presented a larger shielding capability to some commercial glasses [19]. Bektasoglua et al. investigated the mass attenuation coefficients of the ZnO–TeO_2_–Nb_2_O_5_–Gd_2_O_3_ glass system at 20, 30, 40, and 60 keV. In the studied samples, the increasing contribution of Gd_2_O_3_ decreased the value of HVL and increased the value of Z_eff_. At energy 60 keV, glass samples with 1.5, 2, and 2.5 contributions of Gd_2_O_3_ showed greater mass attenuation coefficients than lead [20]. Nazrin et al. studied boro-tellurite glasses doped with copper oxide. That studied glass showed an irregular trend with fluctuations within certain limits of elastic moduli [21]. Fidan et al. examined the radiation shielding ability of TeO_2_–BaO–B_2_O_3_–PbO–V_2_O_5_ glass systems through some shielding parameters. At an energy of 59.5 keV, albedo parameters were also assessed. The intensity of the exposure build-up factor spectra decreased as the mol% of BaO increased. Considering the albedo parameters, BaO represented the maximum reflective compound among the studied glasses [22]. Geidam et al. examined (100 − x) [56TeO_2_ − 24B_2_O_3_ − 20SiO_2_] − xBi_2_O_3_, where x = 0, 1, 2, 3, 4, and 5 mol%, lead-free silica boro-tellurite glasses. With the increase in mol% of Bi_2_O_3_, the nonlinear variation found for the molar volume and density showed. Studied BSBT5 glass with a higher amount of Bi_2_O_3_ mol% displayed supreme values of density and linear attenuation coefficient [23]. Ersundu et al. prepared ZnO-MoO_3_-TeO_2_ glasses. The increase in MoO_3_ on the studied glass systems made them stronger as well as amplified mechanical properties. Studied glass systems displayed lower MFP values comprising concretes and window glass [24]. As a continuation of the previous studies in the TeO_2_-based glasses as promising radiation shielding materials, we selected binary zinc–tellurite glasses and investigated different radiation shielding parameters for the selected glasses.

## 2. Materials and Methods

The elemental composition of the studied samples was obtained from Effendy et al. [25]. The samples are coded as Zn0, Zn5, Zn10, Zn15, Zn20, Zn25, and Zn30 respectively:

Zn0: 0 ZnO–100 TeO_2_ (density = 4.939 g/cm^3^)

Zn5: 5 ZnO–95 TeO_2_ (density = 4.967 g/cm^3^)

Zn10: 10 ZnO–90 TeO_2_ (density = 4.989 g/cm^3^)

Zn15: 15 ZnO–85 TeO_2_ (density = 5.049 g/cm^3^)

Zn20: 20 ZnO–80 TeO_2_ (density = 5.114 g/cm^3^)

Zn25: 25 ZnO–75 TeO_2_ (density = 5.222 g/cm^3^)

Zn30: 30 ZnO–70 TeO_2_ (density = 5.283 g/cm^3^)

The optical characteristics of the glasses would be affected by changes in the composition of the glass forming and glass modifiers. The diverse optical fields are predicted to benefit from the combination of ZnO and tellurite glass networks. Table 1 shows the indirect band gap values. With the help of these, we may glean further information about the optical system. The high concentration of TeO_2_ in these glasses is believed to provide them with exceptional radiation shielding capabilities. Using ZnO as a glass modifier, the optical and gamma-ray shielding capabilities of the TeO_2_ glassy network were examined in this study. The Phy-X/PSD computer software was utilized for determining the shielding properties of glasses against gamma rays [26]. Our prior reports [27,28,29] outline the formulas we used to calculate optical and gamma-ray shielding parameters.

## 3. Results and Discussion

### 3.1. Optical Features

A decrease in the indirect band gap value from 3.515 eV to 3.481 eV was observed once ZnO was introduced into the TeO_2_ network. The tellurite glass network’s regular structure is disrupted by an increase in the network’s non-bridging oxygens (NBOs) when ZnO is added. The structure of the glass will become more erratic as the NBO content rises. Indirect optical band gaps are reduced because the NBO electron bonding is less tight than the bonding oxygen, which weakens the glass networks. One possible explanation for a rise in Zn20’s optical band gap value is that delocalized states are becoming less common, which reduces the number of trap centers. For the remaining optical properties indicated in Table 1, it utilizes the indirect band gap energy estimates.

**Figure 1:**
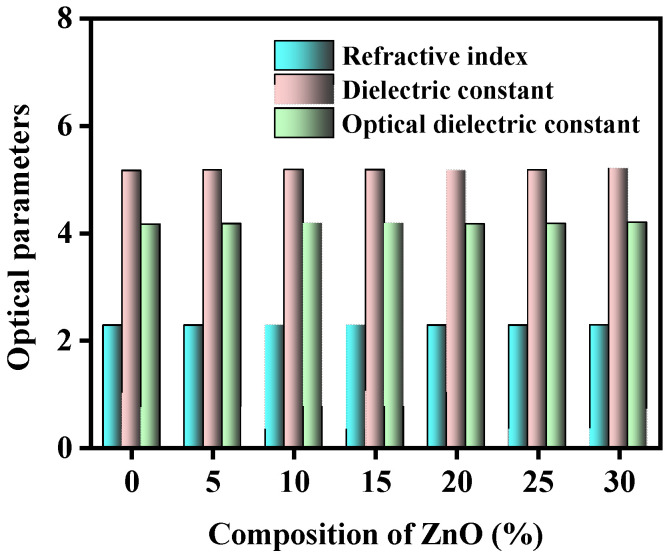
Discrepancy of the refractive index (n), dielectric constant (ε), and optical dielectric constant considering the amount of ZnO.

**Figure 2:**
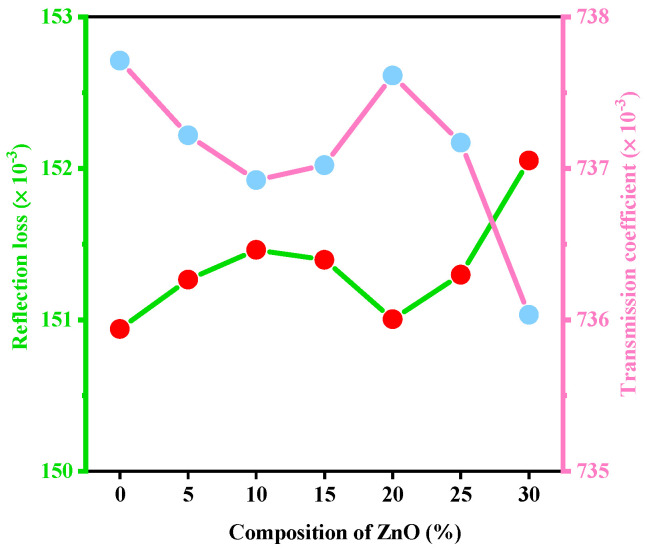
Variation in reflection loss (R_L_) and transmission coefficient (T) considering the amount of ZnO.

**Figure 3:**
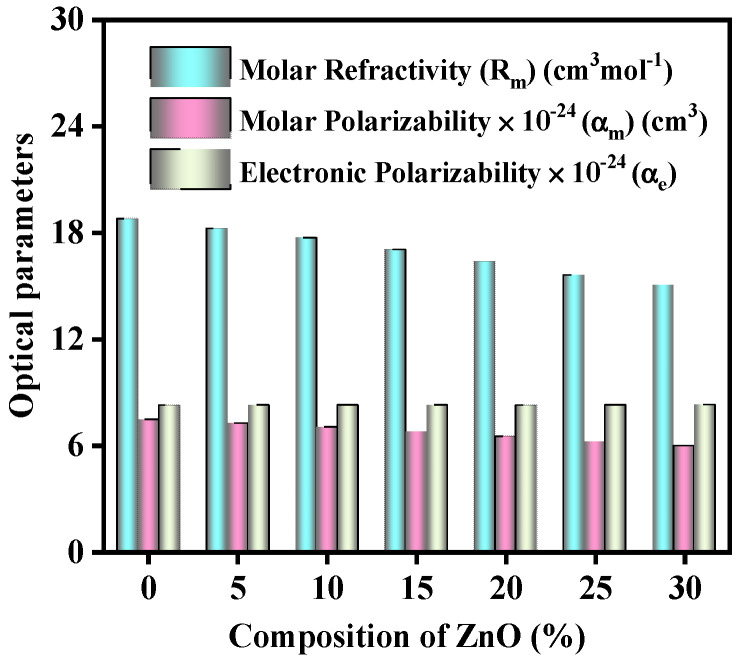
Variation in the molecular polarizability (α_m_), the molecular refractivity (R_m_), and the electronic polarizability (α_e_) considering the amount of ZnO.

**Figure 4:**
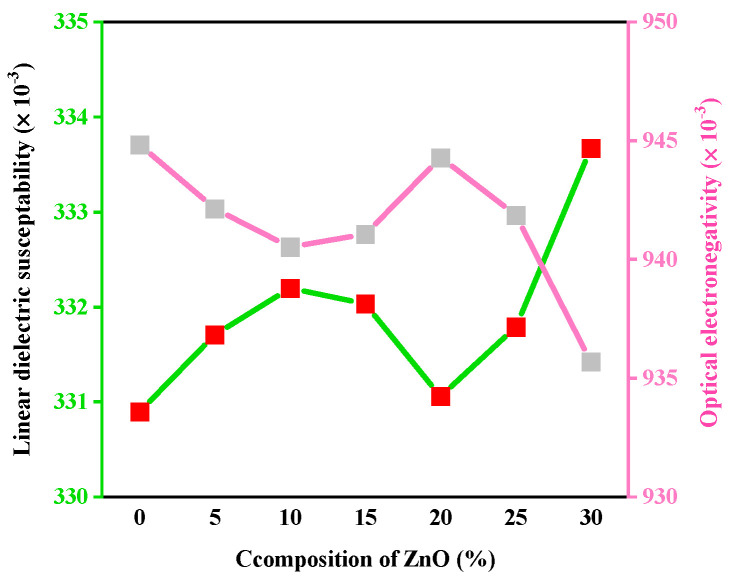
Variation in the linear dielectric susceptibility and optical electronegativity considering the amount of ZnO.

**Figure 5:**
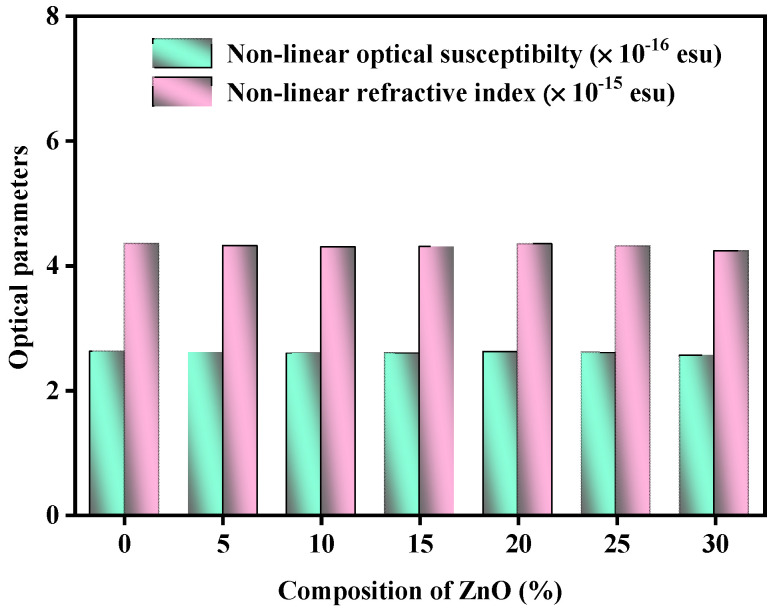
Variation in the non-linear optical susceptibility (χ3) and non-linear refractive index considering the amount of ZnO.

The variation in the several optical properties with the numerous concentrations of ZnO on the studied glass samples has been represented in Figure 1, Figure 2, Figure 3, Figure 4 and Figure 5.

Figure 1 has shown the difference in the refractive index (n), ε and optical dielectric constant considering the amount of ZnO. The n, ε, and optical dielectric constant ($p\frac{dp}{dt})$ values for the increase in NBOs in the network, increase from 2.271–2.278, 5.156–5.191, and 4.156–4.191, respectively, due to the causes of adding ZnO to the samples.

The variation in reflection loss (RL) and transmission coefficient (T), considering the amount of ZnO on the studied glass systems, is displayed in Figure 2. The ZnO content in the glasses has a negligible effect on the RL and T. The RL reduces as the T grows little, and the other way around.

Figure 3 has displayed the variation in the molecular polarizability (α_m_), the molecular refractivity (R_m_), and the electronic polarizability (α_e_), considering the amount of ZnO. When ZnO concentrations are increased, the values of the molecular polarizability (α_m_) and the molecular refractivity (R_m_) drop from 18.767–15.018 cm^3^/mol and from 7.444 × 10^−24^–5.957 × 10^−24^ cm^3^, correspondingly; while the electronic polarizability (α_e_) rises, from 8.244 × 10^24^–8.273 × 10^24^, this is because the increased ZnO concentration for the glasses causes a rise in NBOs.

The values of metallization (M), energy band gap-based metallization (M (E_g_)), and refractive index-based metallization (M (n)) are essentially constant at values of around 0.151, 0.088, and 0.418, respectively. Clearly, the produced glass structures are non-metallic in nature, since all values are less than one.

Variation in the linear dielectric susceptibility and optical electronegativity considering the amount of ZnO is demonstrated in Figure 4. Due to the increase in the quantity of NBOs with increasing ZnO concentration, the value of χ* comes down through 0.945–0.936, even though χ^(1)^ amplified in the range of 0.331–0.334.

Figure 5 illustrates the variation in the non-linear optical susceptibility (χ^3^) and non-linear refractive index, considering the amount of ZnO. Estimates of the present glass samples’ non-linear optical susceptibility (χ^3^) and non-linear refractive index (n_2_^optical^) decreased from 2.620 × 10^−16^–2.558 × 10^−16^ esu and from 4.348 × 10^−15^–4.230 × 10^−15^ esu, respectively. It is possible that the increased concentration of NBOs in the tested glasses is to blame.

### 3.2. Radiation Shielding Study

**Figure 6:**
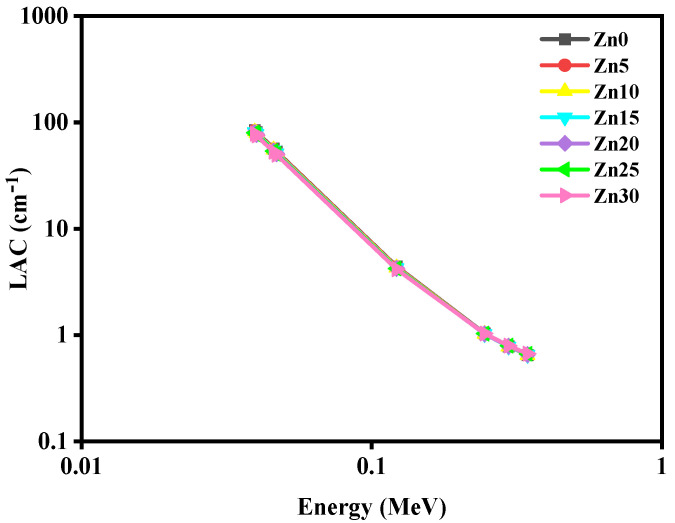
The performance of the linear attenuation coefficients (LAC) of the studied glass samples at studied energies.

Figure 6 reveals the arrangements of linear attenuation coefficients (LAC) according to the incident photon energy of the studied glass samples between the 0.0395–0.344 MeV energy array. In this work, a very low energy of 0.0395–0.344 MeV was considered, which is the difference between the current work and the previous work by Effendy et al. [30]. Between the incident photon energy array of 0.0395–0.3443 MeV, these studied glass samples showed a sharp decrease in the LAC values having energy enhancement.

**Figure 7:**
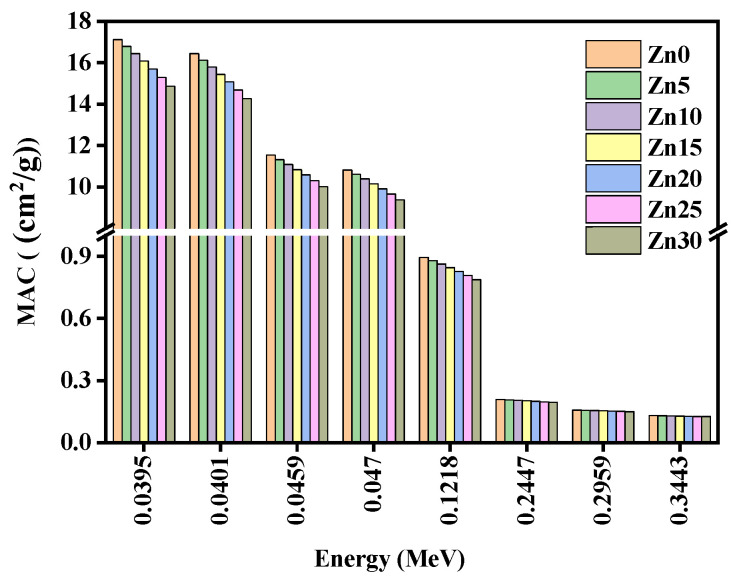
The performance of the mass attenuation coefficients (MAC) of the studied glass samples at studied energies.

Figure 7 demonstrates that the schemes of mass attenuation coefficients (MAC) of the studied glass samples counter the energy range 0.0395–0.3443 MeV. From this figure, it is clear that the evaluated MAC values of the studied glass samples decreased through increasing photon energy. Here, the values of MAC for the studied glass samples followed a very little decreasing trend Zn0 > Zn5 > Zn10 > Zn15 > Zn20 > Zn25 > Zn30 from energy range 0.0395–0.1218 MeV; however, there is a negligible variation obtained from the energy range of 0.2447–0.3443 MeV. The values of MAC presented a greater value at a studied lower energy range from 0.0395–0.047 MeV, yet, showed lesser values at a studied higher energy range from 0.1218–0.3443 MeV. Moreover, the MAC values displayed 1.6 times higher values through enhancing the energy range 0.0395–0.047 MeV; nevertheless, from an energy of 0.047–0.1218 MeV, the MAC values amplified by 8.4 times.

**Figure 8:**
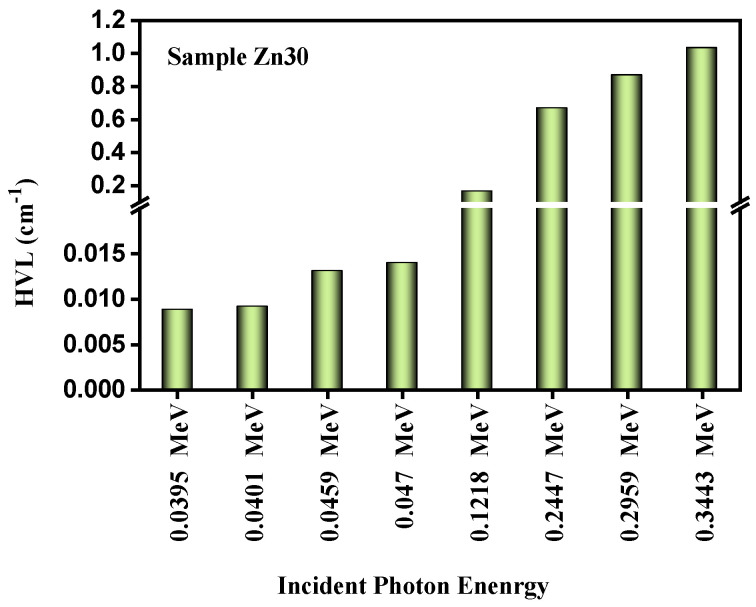
The performance of the HVL of the studied glass samples of Zn30 for the studied energies.

The behavior of the half value layer (HVL) of the studied glass Zn30 sample is shown in Figure 8 against photon energy. This figure reveals an increasing value of half value layer through enhancing photon energy from 0.0395–0.3443 MeV. However, considering the studied glass sample, it was observed that the HVL for the studied sample of Zn30 was enhanced by 117 times higher values from an energy of 0.0395 MeV to 0.3443 MeV.

**Figure 9:**
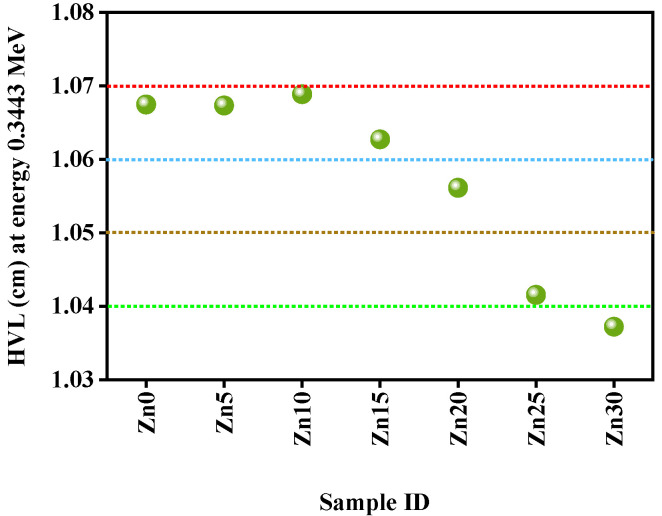
Presentation of the half value layer of all studied glass samples at an energy of 0.3443 MeV.

Figure 9 shows the value of the HVL of all studied glass samples at an energy of 0.3443 MeV. It is clear that the Zn10 sample displayed the greatest value of 1.068 cm and the Zn30 sample revealed the lowest value of 1.037 cm, which exemplifies that sample Zn30 has the compositional glass among the studied glass samples for radiation shielding.

**Figure 10:**
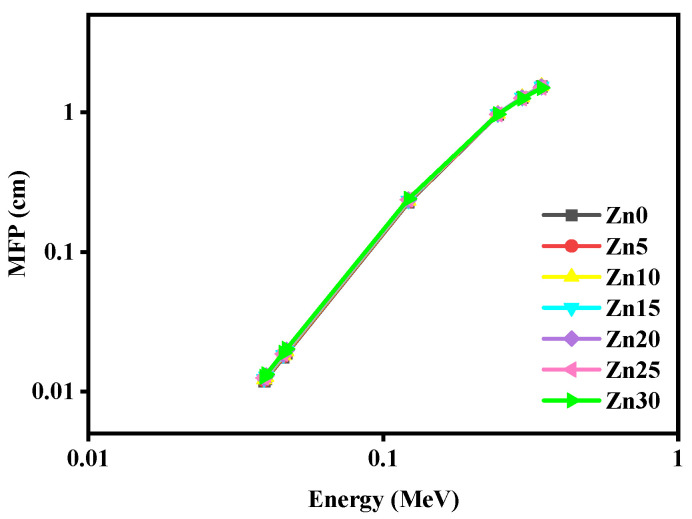
The performance of the MFP of the studied samples for the studied energies.

Figure 10 illustrates the MFP deeds of studied glass samples at the energy range 0.0395–0.3443 MeV. The MFP of the considered glass samples has displayed an increasing attitude through the intensification of incident photon energy.

This suggests that a thin layer of Zn-X glasses is needed to shield the low-energy photons, while when the energy of the radiation is increased; we need a thicker glass to enhance the shielding performance of the glass. This is in line with the recent results for other glass systems [31,32,33]. Additionally, we found that the addition of ZnO has an impact on the MFP. Since the addition causes an increase in the density from 4.939 to 5.283 g/cm^3^, then the MFP slightly decreases and this is acceptable according to the inverse relation between the MFP and the density of the shield.

**Figure 11:**
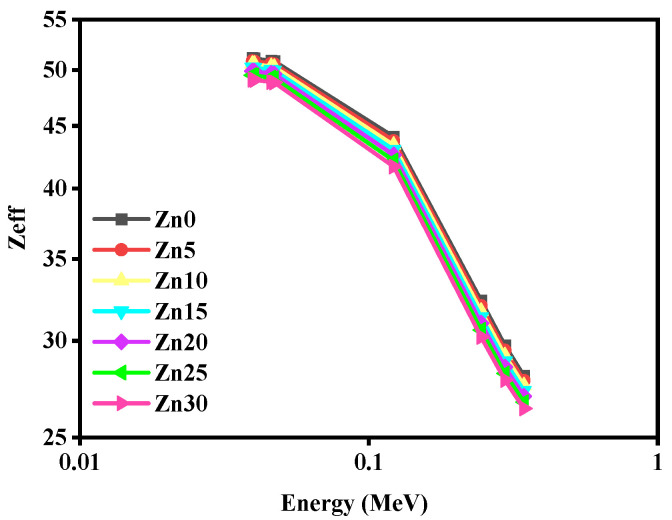
The performance of the effective atomic number of the studied glass samples for the studied energies.

Figure 11 portrays the discrepancy of the Z_eff_ of the studied glass samples through the photon energy range 0.0395–0.3443 MeV. It has been obtained that the Z_eff_ of the studied glass samples has decreased through enhancing photon energy. Plainly, a sharp declination has been demonstrated for the values of Z_eff_ of the studied glass samples from the energy range 0.1218–0.3443 MeV.

**Figure 12:**
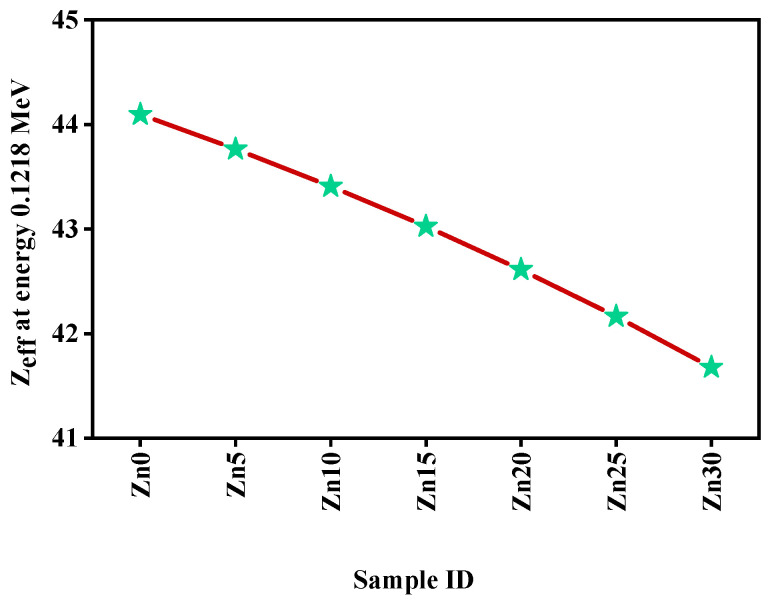
The performance of the Z_eff_ for all studied glass samples at energy 0.1218 MeV.

The value of the Z_eff_ of all the considered samples has been demonstrated for an energy of 0.1218 MeV (Figure 12). Sample Zn30 showed the minimum value of Zeff likening to other samples because of the lower amount of TeO_2_, conclusively, the studied glass Zn0 sample appeared to be the maximum value of Zeff among the other studied samples.

## 4. Conclusions

The radiation defensive efficiency and the optical features of the TeO_2_ glass matrix were analyzed considering the effect of the concentration of the ZnO modifier. The introduction of ZnO into the TeO_2_ network results in a decrease in the value of the indirect band gap, which goes from 3.515 to 3.481 eV. The values of the refractive index, dielectric constant, and optical dielectric constant all increased between the ranges 2.271–2.278, 5.156–5.191, as well as 4.156–4.191, accordingly. Transmission coefficient and reflection loss operate in a manner that is opposed to one another. With an increase in ZnO concentration in the selected glasses, the values of molar refractivity and molar polarizability decrease from 18.767 cm^3^/mol to 15.018 cm^3^/mol and from 7.444 × 10^−24^ cm^3^ to 5.957 × 10^−24^ cm^3^, respectively. On the other hand, the electronic polarizability increases from 8.244 × 10^24^ to 8.273 × 10^24^. The metallization values support that the glass systems have a non-metallic fundamental composition. Regarding the values of LAC, MAC, HVL, MFP, and Z_eff_, the Zn30 sample showed the best radiation shielding ability comprising other studied samples.

## Figures and Tables

**Table 1:** UV parameters of present glasses.

Properties	Glass Samples						
	Zn0	Zn5	Zn10	Zn15	Zn20	Zn25	Zn30
The indirect band gap (eV)	3.515	3.505	3.499	3.501	3.513	3.504	3.481
n	2.271	2.273	2.274	2.274	2.271	2.273	2.278
ε	5.156	5.166	5.172	5.170	5.158	5.167	5.191
** $p\frac{dp}{dt}$ **	4.156	4.166	4.172	4.170	4.158	4.167	4.191
R_L_	0.151	0.151	0.151	0.151	0.151	0.151	0.152
T	0.738	0.737	0.737	0.737	0.738	0.737	0.736
R_m_ (cm^3^/mol)	18.767	18.223	17.698	17.033	16.352	15.593	15.018
α_m_ × 10^−24^ cm^3^	7.444	7.228	7.0203	6.756	6.486	6.185	5.957
α_e_ × 10^24^	8.244	8.252	8.257	8.256	8.246	8.253	8.273
M	0.151	0.151	0.151	0.151	0.151	0.151	0.152
M (E_g_)	0.088	0.088	0.087	0.088	0.088	0.088	0.087
M (n)	0.419	0.419	0.418	0.418	0.419	0.419	0.417
χ^(1)^	0.331	0.332	0.332	0.332	0.331	0.332	0.334
χ*	0.945	0.942	0.941	0.941	0.944	0.942	0.936
χ^3^ × 10^−16^ (esu)	2.620	2.602	2.591	2.594	2.616	2.600	2.558
n_2_^optical^ × 10^−15^ (esu)	4.348	4.313	4.292	4.299	4.341	4.310	4.230

## Data Availability

Not applicable.
